# Diagnostic performance of conventional ultrasound and quantitative and qualitative real-time shear wave elastography in musculoskeletal soft tissue tumors

**DOI:** 10.1186/s13018-020-01620-x

**Published:** 2020-03-11

**Authors:** Ao Li, Xiao-Jing Peng, Qian Ma, Ye Dong, Cui-Lian Mao, Yu Hu

**Affiliations:** Department of Ultrasound, the First Affiliated Hospital of Nanjing Medical University, Nanjing, 210029 China

**Keywords:** Musculoskeletal soft tissue tumor, Ultrasound, Real time, Shear wave elastography

## Abstract

**Background:**

To explore the feasibility to identify malignant musculoskeletal soft tissue tumors using real-time shear wave elastography (rtSWE).

**Methods:**

One hundred fifteen musculoskeletal soft tissue tumors in 92 consecutive patients were examined using both conventional ultrasonography (US) and rtSWE. For each patient, the rtSWE parameters including maximum elasticity (*E*_max_), mean elasticity (*E*_mean_), minimum elasticity (*E*_min_), standard deviation of the elasticity (*E*_sd_), and rtSWE image pattern were obtained. Eighty-one histopathologically confirmed tumors from 73 patients were subjected to analysis.

**Results:**

The 81 lesions included in the study were histopathologically classified as malignant (*n* = 21) or benign (*n* = 60). The statistically significant differences between benign and malignant lesions were found in conventional US characters including size, depth, margin, echogenicity, mass texture, and power Doppler signal. Meanwhile, the significant differences were also found in quantitative rtSWE findings including *E*_max_, *E*_mean_, *E*_min_, and *E*_sd_ values and in qualitative rtSWE parameter named rtSWE image pattern. Multivariate analysis showed that infiltrative margin (OR, 4.470), and size (OR, 1.046) were independent predictors for malignancy in US findings, while *E*_sd_ value (OR, 9.047) was independent predictors for malignancy in quantitative rtSWE parameters. Areas under the ROC curve (Azs) for US features, E_sd_ value, and rtSWE image pattern were 0.851, 0.795, and 0.792, respectively.

**Conclusions:**

Conventional US and quantitative and qualitative rtSWE parameters are useful for malignancy prediction of musculoskeletal soft tissue tumors. rtSWE can be used to supplement conventional US to diagnose musculoskeletal soft tissue tumors.

## Background

Musculoskeletal soft tissue tumors are a heterogeneous group including tumor-like lesions, benign, and malignant tumors [1] with a benign to malignant ratio of over 100:1 [2]. An overall incidence of 300 cases per 100,000 population has been reported [3]. Whereas it is essential to accurately identify the potentially malignant lesions for timely therapy, the imaging evaluation has challenges due to the considerable heterogeneity of pathological tissues of the sarcomas [1].

Ultrasonography (US) has a high sensitivity in the diagnosis of benign and malignant soft tissue tumors that has been widely used in clinical application [4, 5]. The advantages of US include its good spatial and contrast resolution, real-time imaging capability, and ability to assess tissue vascularity. But regarding to its operator dependence, limited awareness of some specific tumors and their ultrasound features may reduce the diagnostic accuracy [6]. Thus, different imaging modalities have been employed together to assess soft tissue tumors, such as magnetic resonance imaging (MRI), computed tomography (CT), and positron emission tomography-CT (PET-CT). However, none of these approaches are reliable enough for the diagnosis of all soft tissue tumors. CT is advantageous to evaluate four distinct characteristics of musculoskeletal soft tissue tumors: lesion density, mineralization patterns, bone involvement, and vascular involvement; but it is not sensitive and can not demonstrate the relationship of the mass and the adjoining neurovascular structures [7]. PET-CT provides the anatomic correlation and information about metabolic activity of a soft tissue tumor; therefore, it is not used for initial evaluation [8]. Although MRI offers good contrast resolution for soft tissue elements such as fat, muscle, and bone, no single MRI feature can be reliably used to give an accurate diagnosis of all soft tissue tumors. In particular, some benign masses related to inflammatory, traumatic, or degenerative processes may have common characteristics of MRI signal intensity with malignant tumors [9]. Thus, the diagnostic accuracy of these imaging modalities in the assessment of soft tissue tumors still needs to be improved.

Tissue stiffness is an important parameter in diagnosing potentially malignant tissue or other diseased tissue. Previously, the differential diagnosis was primarily based on palpations by the clinical doctors, which was indirect and could be limited in patients with large lesions, obesity, and doctors’ experiences. Thus, the elastography techniques were developed to meet these challenges. Currently, there are several elastography techniques depending on the type of stress application and the method used to detect tissue displacement and construct the image. The main techniques used in the clinical practice include strain elastography, shear wave elastography (SWE), transient elastography, and acoustic radiation force elastography. Each technique is referred to a variety of different names in the literature and in commercially available systems. Real-time shearwave™ elastography (rtSWE or SWE™) is a relatively novel technique, pioneered by SuperSonic Imagine, which allows physicians to visualize and quantify the stiffness of tissue in a real-time, reliable, and reproducible manner. It has already been widely accepted as an effective method for lesion detection and characterization in the liver, breast, thyroid, and prostate, which has been shown to be clinically viable both qualitatively and quantitatively with high reproducibility [10–12].

To the best of our knowledge, there is no report in literature about the application of rtSWE in the differentiation of musculoskeletal soft tissue tumors. As the elastic properties of the tissue structure changes with the pathologic development [13], we decided to assess whether the quantitative and qualitative rtSWE could be applied in the differential diagnosis of musculoskeletal soft tissue tumors.

## Materials and methods

### Patients

The study was approved by the institutional ethics, and all patients underwent oral informed consent. One hundred fifteen soft tissue tumors of extremity and trunk in 92 consecutive patients were examined using both conventional ultrasonography and SWE for palpable masses from September 2016 to January 2020. All masses subsequently underwent ultrasonic-guided biopsy, 65 of which were excised by surgery on basis of the biopsy result. Histopathological evaluation with the biopsy or excision specimen (where available) was used as the gold standard. The operations were performed by two senior orthopedic surgeons. The pathologic diagnosis was confirmed by one soft tissue tumor pathological specialist. Masses without definite histopathological result were excluded. Among these, 81 masses in 73 patients including 30 men and 43 women (age range 11~84 years, mean 43.9 ± 18.1 years) were included in this analysis. The histopathologic diagnosis of the benign tumors (*n* = 60) included localized tenosynovial giant cell tumor (*n* = 4), pseudosarcomatousfasciitis (*n* = 2), schwannoma (*n* = 12), fibrous histiocytoma (*n* = 2), pilomatrixoma (*n* = 2), fibroma (*n* = 1), hemangioma (*n* = 9), angioleiomyoma (*n* = 5), lipomas (*n* = 20), elastofibroma (*n* = 1), and neurofibroma (*n* = 2). Malignant tumors (*n* = 21) included metastatic carcinoma (*n* = 2), myxoid liposarcoma (*n* = 3), plexiform fibrohistiocytic tumor (*n* = 1), myxofibrosarcoma (*n* = 1), synovial sarcoma (*n* = 2), rhabdomyosarcoma (*n* = 1), fusocellular sarcoma (*n* = 4), lymphoma (*n* = 2), solitary fibrous tumor (*n* = 1), malignant mesenchymoma (*n* = 2), and undifferentiated sarcoma (*n* = 2).

### US examinations and image evaluation

All US and rtSWE examinations were obtained with a US system (Aixplorer; SuperSonic Imagine, Aix en Provence, France) equipped with a 4–15 MHz liner transducer and a 1–6 MHz convex transducer by two sonographers who had more than 3 years of experience in musculoskeletal ultrasound. US features of each mass including size, depth (superficial or deep to deep fascia), margins (well-defined rim or infiltrative), echogenicity (hyperechogeneity, isoechoic, or hypoechogeneity), mass texture (heterogeneous or homogeneous), and power Doppler signal (absent, linear, or disorganized) were evaluated in real time. And then, rtSWE followed. The display presented elastograms overlaid on gray-scale images, setting the region of interest (ROI) so that it included the lesion and the surrounding normal tissue. Tumors whose size exceeded the maximum ROI (6.5 × 4 cm) were excluded from this study. rtSWE was conducted with the aid of a movable intelligent unit displaying tissue stiffness on a color scale; progression from blue to red indicates increasing shear modulus (stiffness). The color map scale ranged from 0 to 600 kPa. rtSWE images of the masses were saved after a few seconds of immobilization to allow the rtSWE image to stabilize. Each subject was obtained three reliable rtSWE images to take the average of measurements as the result. The rtSWE images were classified into four patterns by the visual evaluation [14]: coded blue homogeneously (pattern 1), vertical stripe pattern artifacts (pattern 2), a localized colored area at the margin of the lesion (pattern 3), and heterogeneously colored areas in the interior of the lesion (pattern 4). Quantitative elasticity was measured on the rtSWE images using the system’s quantification tool, known as the Q-Box. The minimum, maximum, and mean elasticity values in terms of the Young modulus (in kilopascals) and SD were measured in the mass including immediate adjacent stiff tissue or halo.

### Statistical analysis

Statistical Product and Service Solutions software (SPSS version 17.0 software IBM Corporation, Armonk, NY USA) was used for the statistical analysis. Margin irregularities, echogenicity, power Doppler signal, and the rtSWE color patterns were compared between the benign tumors and malignant tumors using Fisher’s exact test. The quantitative rtSWE parameters and tumor size were compared using Mann–Whitney *U* tests. Findings were considered statistically significant at *p* < 0.05. Malignancy risks for independent variables, odds ratios (ORs), and 95% confidence intervals (95% CIs) for each feature were calculated with multivariate logistic regression analysis. Receivers operating characteristic (ROC) curve was used to evaluate the diagnostic performances of US features, quantitative rtSWE parameters, and qualitative rtSWE parameters in differentiating malignant from benign tumors. Areas under the ROC curves (Az) were calculated and then compared by z test.

## Results

### Conventional US

**Fig. 1:**
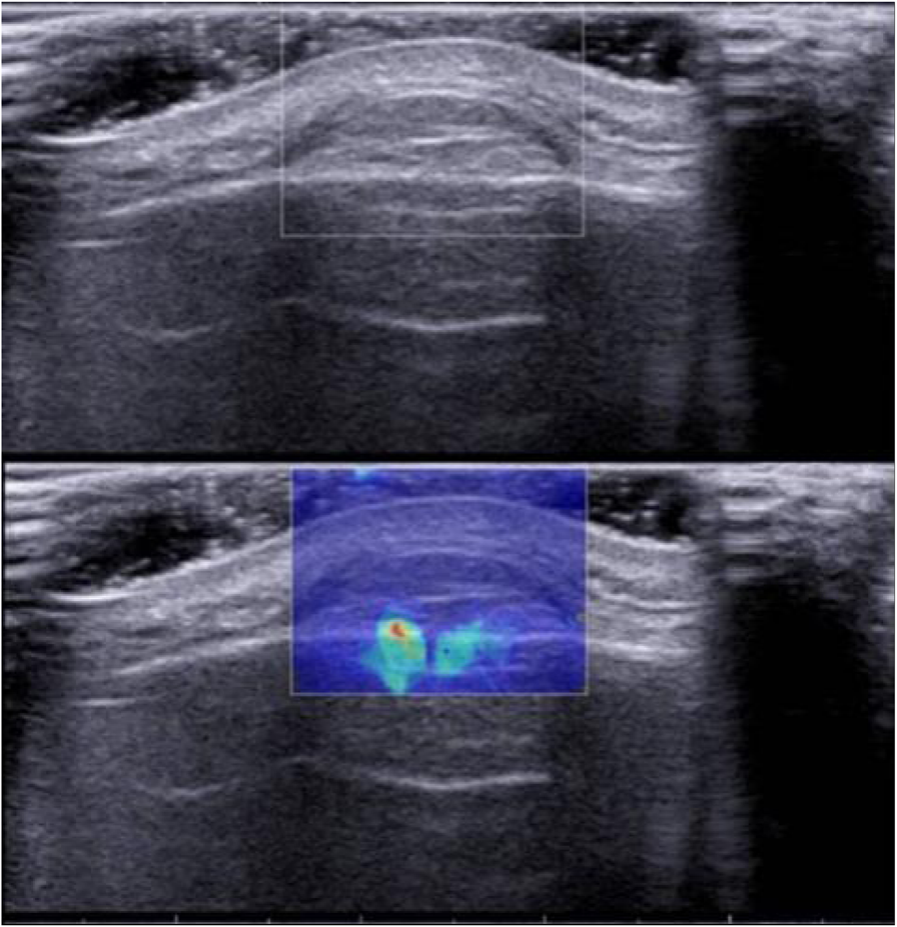
Lipoma in the forehead of a 41-year-old man. US image showed the tumor was superficial to deep fascia, isoechoic, homogeneous, and well-defined in margin. SWE image shows coded blue homogeneously (pattern 1)

**Fig. 2:**
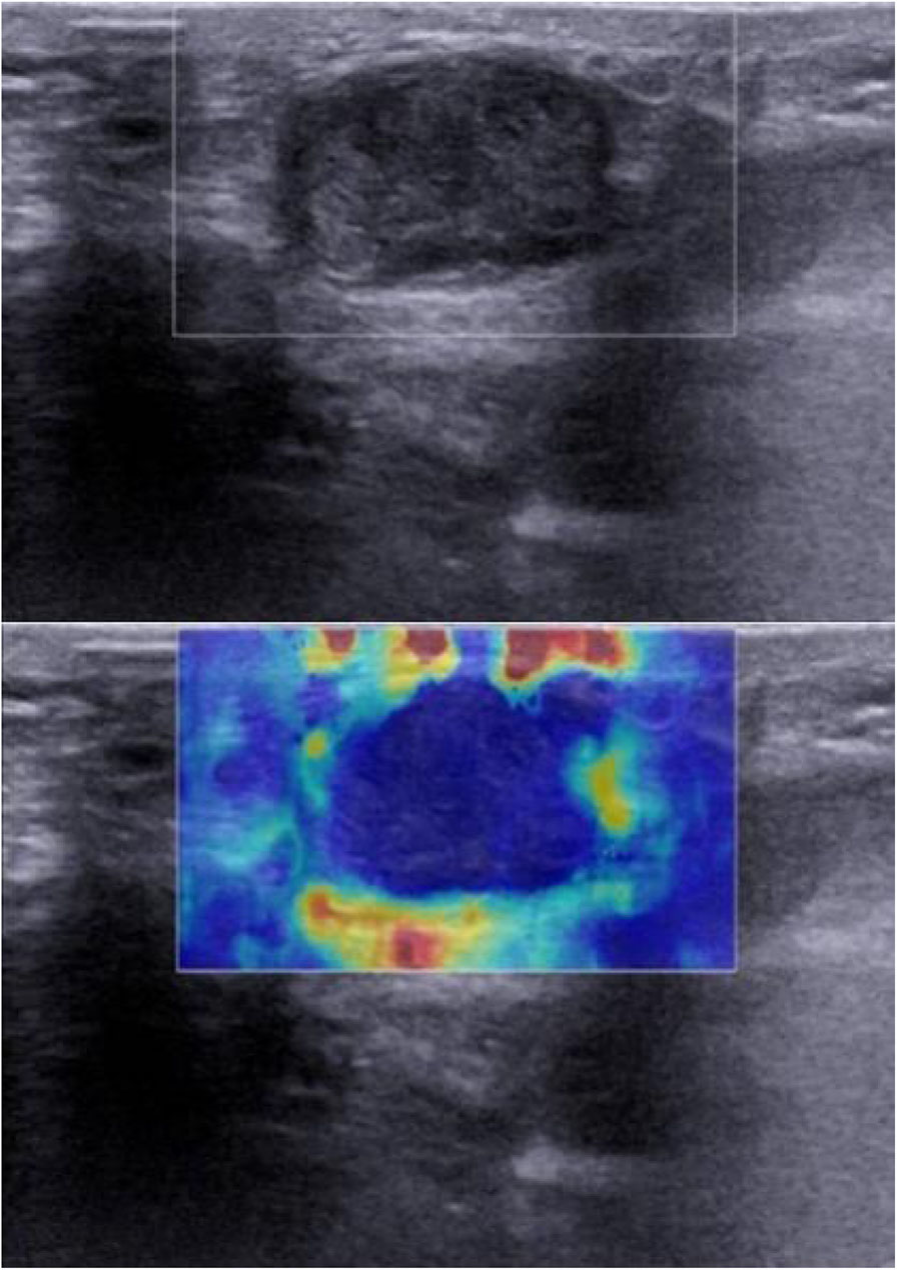
Rhabdomyosarcoma in the thigh of a 56-year-old woman. US image showed the tumor was deep to deep fascia, hypoechoic, heterogeneous, and infiltrative in margin. SWE image shows a localized colored area at the margin of the lesion (pattern 3)

**Fig. 3:**
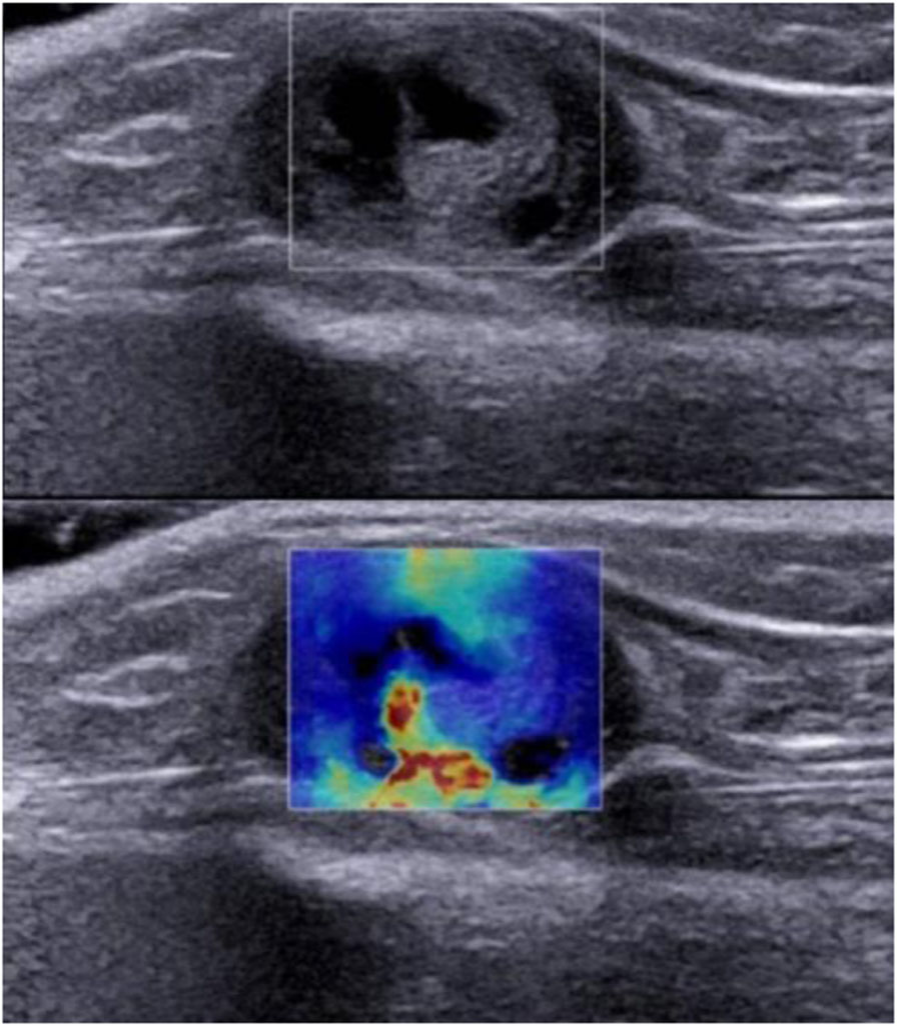
Schwannoma of the ulnar nerve in a 54-year-old woman. US image showed the tumor was deep to deep fascia, hypoechoic, heterogeneous, and well-defined in margin. SWE image shows heterogeneously colored areas in the interior of the lesion (pattern 4)

In this study, on conventional ultrasonography, the benign tumors frequently appeared as superficial to deep fascia (37/60, 61.7%) and hypoechoic (38/60, 63.3%) lesions with heterogeneous echogenicity (35/60, 58.3%), well-defined rim (54/60, 90%), and disorganized power Doppler signal (25/60, 41.7%); while the malignant tumors frequently appeared as deep to deep fascia (14/21, 66.7%) and hypoechoic (21/21, 100%) lesions with heterogeneous echogenicity (18/21, 85.7%), well-defined rim (12/21, 57.1%), and disorganized power Doppler signal (13/21, 61.9%) (Table 1, Figs. 1, 2, and 3).

**Table 1:** US characteristics of benign and malignant tumors

	Size (mm) *M* (*Q*_*R*_)	Depth superficial to deep fascia	Margin well-defined rim (%)	Echogenicity hypo/iso/hyper (%)	Mass texture heterogeneous (%)	Power Doppler signal absent/linear/disorganized (%)
Benign tumor (*n* = 60)	26 (14~42)	37 (61.7)	54 (90.0)	38/12/10 (63.3/20.0/16.7)	35 (58.3)	20/15/25 (33.3/25.0/41.7)
Malignant tumor (*n* = 21)	45 (26~67.5)	7 (33.3)	12 (57.1)	21/0/0 (100/0/0)	18 (85.7)	1/7/13 (4.8/33.3/61.9)
*p* value	< 0.001	0.041	0.002	0.002	0.032	0.024

US findings including size, depth, margin, echogenicity, mass texture, and power Doppler signal were all significantly different in benign and malignant musculoskeletal soft tissue tumors (*p* < 0.05) (Table 1). By multivariate logistic regression analysis, margin (OR 4.470; 95% CI 1.036, 19.277) was the strongest independent predictor for malignancy, followed by size (OR 1.046; 95% CI 1.013, 1.080) (Table 2).

**Table 2:** Multivariate logistic regression analysis for US features

Characteristics	*β*	SE	OR	95% CI	*p* value
Margin	1.497	0.746	4.470	1.036~19.277	0.045
Size	0.045	0.016	1.046	1.013~1.080	0.006

*β* regression coefficient, *SE* standard error

The associated Az was 0.851 (95% CI 0.754, 0.920) with a malignancy predictive model including the two variables (margin and size). When the cut-off value for prediction was 0.338, the sensitivity, specificity, positive predictive value (PPV), negative predictive value (NPV), and accuracy were 71.4%, 86.7%, 65.2%, 89.7%, and 75.4%, respectively (Table 4).

### rtSWE findings

Quantitative rtSWE parameters including *E*_max_, *E*_mean_, *E*_min_, and *E*_sd_ values were all significantly different in benign and malignant musculoskeletal soft tissue tumors (*p* < 0.05) (Table 2). By multivariate logistic regression analysis, *E*_sd_ value (OR 9.047; 95% CI 2.367, 34.583) was the strongest independent predictor for malignancy. Qualitative rtSWE parameters, known as rtSWE image patterns, were significantly different between benign and malignant musculoskeletal soft tissue tumors (*p* < 0.05) (Table 3, Figs. 1, 2, and 3).

**Table 3:** rtSWE characteristics of benign and malignant tumors

	*E*_max_ (m/s) *M* (*Q*_*R*_)	*E*_mean_ (m/s) *M* (*Q*_*R*_)	*E*_min_ (m/s) *M* (*Q*_*R*_)	*E*_sd_ (m/s) *M* (*Q*_*R*_)	SWE image pattern I/II/III/IV (%)
Benign tumor	3.8 (3.00~5.15)	2.40 (1.90~3.75)	1.40 (0.80~2.50)	0.42 (0.29~0.68)	37/17/1/5 (61.7/28.3/1.7/8.3)
Malignant tumor	5.76 (3.55~7.20)	3.20 (2.35~4.25)	0.50 (0.20~1.20)	0.88 (0.61~1.49)	4/4/2/11 (19.0/19.0/9.5/52.4)
*p* value	< 0.001	0.001	< 0.001	< 0.001	< 0.001

The Az was 0.795 (95% CI 0.691, 0.877) for malignancy prediction with *E*_sd_ value. When the cut-off value was 0.8, the sensitivity, specificity, PPV, NPV, and accuracy were 66.7%, 85.0%, 60.9%, 87.9%, and 71.4%, respectively. The associated Az was 0.792 (95% CI 0.687, 0.874) for malignancy prediction with rtSWE image patterns. When the cut-off value was pattern III or IV, the sensitivity, specificity, PPV, NPV, and accuracy were 61.9%, 90.0%, 68.4%, 87.1%, and 69.2%, respectively (Table 4).

**Table 4:** The diagnostic performance of US feature predictive model, *E*_sd_ value, and rtSWE image pattern for malignancy prediction

Methods	Optimized cut-off value	Sensitivity (%)	Specificity (%)	PPV (%)	PNV (%)	Accuracy (%)	Az (95% CIs)
US feature predictive model	Pre value > 0.338	71.43	86.7	65.2	89.7	75.4	0.851 (0.754~0.920)
*E*_sd_ value	> 0.8	66.7	85	60.9	87.9	71.4	0.795 (0.691~0.877)
rtSWE image pattern	Pattern III or IV	61.9	90.0	68.4	87.1	69.2	0.792 (0.687~0.874)

**Fig. 4:**
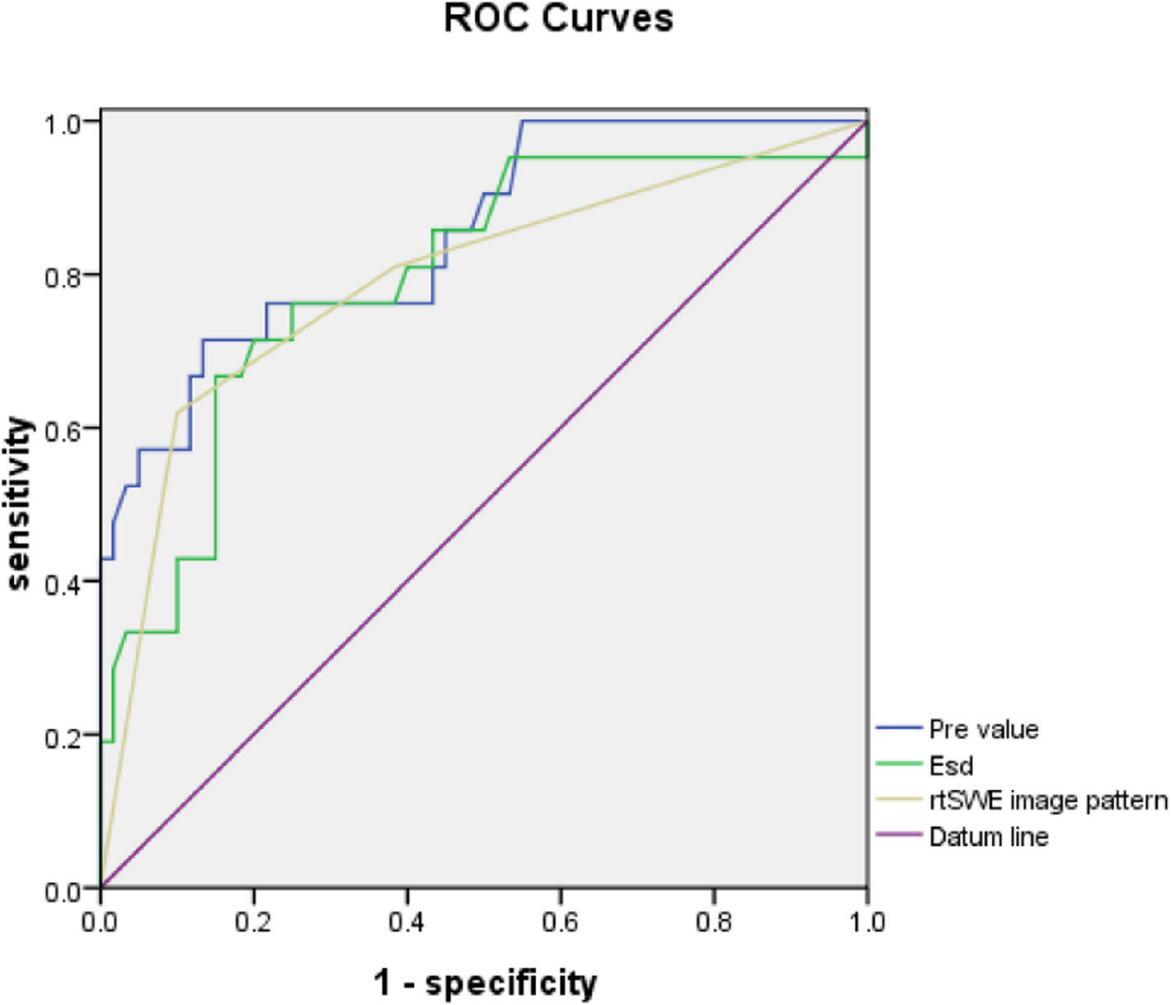
ROC curves for US feature predictive model, *E*_sd_ value, and rtSWE image pattern. Azs for them are 0.851, 0.795, and 0.792, respectively

Comparing the Azs of the US feature predictive model, quantitative rtSWE parameters, and qualitative rtSWE parameters, there were no significant differences between any two of them (all *p* ≥ 0.05) (Fig. 4).

## Discussion

In conventional US, margin was revealed to be the strongest independent predictor for malignant musculoskeletal soft tissue tumors (OR 4.470), followed by size (OR 1.046). Tumor margin has been frequently referred to as a useful parameter for detecting malignant soft tissue tumors [15, 16]. It was thought that the incidence of an infiltrative margin was larger in malignant tumors than benign tumors [16]. This trend was found in our own study. An infiltrative margin was present in 6 out of 60 benign and 9 out of 21 malignant cases. Oebisu et al. [5] reported that tumor size was significant different in benign and malignant soft tissue tumors, which is consistent with the results of our present study. Lesion size greater than or equal to 5 cm was regarded as significant risk factor for malignant soft tissue tumors [17]. Other US parameters such as depth, echogenicity, mass texture, and power Doppler signal were also different in benign and malignant soft tissue tumors in the present study, but they were not included in the multivariate logistic regression equation. US feature was found helpful in malignancy prediction for musculoskeletal soft tissue tumors with an associated Az of 0.851. The diagnostic efficacy was considered as moderate (0.7 < Az ≤ 0.9).

Ultrasonic elastography, a technique that provides the information regarding tissue stiffness, may be an important supplement to the morphologic evaluation. Hahn et al. [18] evaluated the value of strain elastography for differentiation of benign and malignant soft tissue tumors and demonstrated the strain ratio as a diagnostic indicator to predict the malignant potential. Unlike the strain elastography, rtSWE visualizes the tissue elasticity with no requirement of ultrasound transducer compression; therefore, it is an excellent quantitative and qualitative elastography with high reproducibility. Pass et al. [2] reported that there was no statistically significant association between longitudinal velocity and malignancy, but some evidence showed that higher transverse velocity was associated with decreased odds of malignancy. Likewise, the quantitative component of our study revealed no significant difference in *E*_max_ values between malignant and benign tumors, as well as *E*_mean_ values. But there were significant differences in *E*_min_ and *E*_sd_ values between the two groups. Malignant tumors showed a more heterogeneous nature with hemorrhage or necrosis in the interior of the lesions than did benign tumors (Figs. 1 and 2), which may account for the significant trend for malignant tumors to exhibit lower *E*_min_ and higher *E*_sd_ values. *E*_sd_ value was the strongest independent predictor for malignancy in quantitative rtSWE parameters (OR 9.047). The associated Az was 0.795 for malignancy prediction with *E*_sd_ value.

Tozaki and Yoon et al. classified the image patterns visually of SWE images for differential diagnosis between benign and malignant solid breast masses [14]. It was considered that if the measurement conditions were made uniform, SWE images of different lesions could be compared. We chose a protocol for musculoskeletal general in this study with the color map scale ranged from 0 to 600 kPa and found that rtSWE image patterns correlated with the histopathologic composition of the soft tissue tumors; malignancy was found to be more likely in pattern 3 and 4. It was noted that malignant lesions were surrounded by a halo that represented a desmoplastic reaction of tissue to tumor infiltration [19]. Furthermore, heterogeneously colored areas in the tumor represented that both high and low elasticity values existed, similar to the quantitative rtSWE. The associated Az was 0.792 for malignancy prediction with rtSWE image patterns.

The diagnostic efficacy was moderate in the quantitative and qualitative rtSWE parameters and the US feature predictive model, with no significant differences between any two of them. Overall, rtSWE was an important supplementary to ultrasound in musculoskeletal soft tissue tumor diagnosis.

The current study has some limitations. First, bias of case selection may be occurred in this study, due to the large number of histologically distinct entities of soft tissue tumors [20]. Larger patient cohorts are necessary to be researched for improving the diagnostic efficiency and accuracy of conventional US and rtSWE parameters. Secondly, the default maximum display setting of 600 kPa was used in the color map scale in this study; the relative high scale may reduce the color distinction in rtSWE color pattern. More practices are needed to confirm its appropriateness. Lastly, we just compared diagnostic performance of conventional US and rtSWE; a combination of rtSWE with conventional US or MRI could be promising in the further research.

## Conclusions

The rtSWE technique could be used as a noninvasive method for obtaining information regarding tissue stiffness for evaluating musculoskeletal soft tissue tumors. US features and quantitative and qualitative rtSWE parameters were illuminated to be useful for malignancy prediction of musculoskeletal soft tissue tumors.

## Data Availability

The datasets used and analyzed during the current study are available from the corresponding author on reasonable request.

## Abbreviations

95% CI: 95% confidence interval
Az: Areas under the ROC curve
CT: Computed tomography
*E*_max_: Maximum elasticity
*E*_mean_: Mean elasticity
*E*_min_: Minimum elasticity
*E*_sd_: Standard deviation of the elasticity
MRI: Magnetic resonance imaging
NPV: Negative predictive value
OR: Odds ratio
PET-CT: Positron emission tomography-CT
PPV: Positive predictive value
ROC: Receivers operating characteristic
ROI: Region of interest
rtSWE: Real-time shear wave elastography.
SPSS: Statistical Product and Service Solutions
US: Ultrasonography
